# Medical resident’s pursuing specialty and differences in clinical proficiency among medical residents in Japan: a nationwide cross-sectional study

**DOI:** 10.1186/s12909-023-04429-4

**Published:** 2023-06-22

**Authors:** Takashi Watari, Yuji Nishizaki, Nathan Houchens, Koshi Kataoka, Kota Sakaguchi, Yoshihiko Shiraishi, Taro Shimizu, Yu Yamamoto, Yasuharu Tokuda

**Affiliations:** 1 General Medicine Center, Shimane University Hospital, 89-1, Enya-Cho, Izumo Shi, Shimane, 693-8501 Japan; 2 Medicine Service, VA Ann Arbor Healthcare System, Ann Arbor, MI USA; 3 Department of Internal Medicine, University of Michigan Medical School, Ann Arbor, MI USA; 4 Division of Medical Education, Juntendo University School of Medicine, Tokyo, Japan; 5 Department of Diagnostic and Generalist Medicine, Dokkyo Medical University Hospital, Tochigi, Japan; 6 Division of General Medicine, Center for Community Medicine, Jichi Medical University, Tochigi, Japan; 7 Muribushi Okinawa Project for Teaching Hospitals, Okinawa, Japan

**Keywords:** Postgraduate medical education, Essential clinical skills, General medicine, Cross-sectional study

## Abstract

**Importance:**

Standardized examinations assess both learners and training programs within the medical training system in Japan. However, it is unknown if there is an association between clinical proficiency as assessed by the General Medicine In-Training Examination (GM-ITE) and pursuing specialty.

**Objective:**

To determine the relative achievement of fundamental skills as assessed by the standardized GM-ITE based on pursuing career specialty among residents in the Japanese training system.

**Design:**

Nationwide cross-sectional study.

**Setting:**

Medical residents in Japan who attempted the GM-ITE in their first or second year were surveyed.

**Participants:**

A total of 4,363 postgraduate years 1 and 2 residents who completed the GM-ITE were surveyed between January 18 and March 31, 2021.

**Main measures:**

GM-ITE total scores and individual scores in each of four domains assessing clinical knowledge: 1) medical interview and professionalism, 2) symptomatology and clinical reasoning, 3) physical examination and treatment, and 4) detailed disease knowledge.

**Results:**

When compared to the most pursued specialty, internal medicine, only those residents who chose general medicine achieved higher GM-ITE scores (coefficient 1.38, 95% CI 0.08 to 2.68, *p* = 0.038). Conversely, the nine specialties and “Other/Not decided” groups scored significantly lower. Higher scores were noted among residents entering general medicine, emergency medicine, and internal medicine and among those who trained in community hospitals with higher numbers of beds, were more advanced in their training, spent more time working and studying, and cared for a moderate but not an extreme number of patients at a time.

**Conclusions:**

Levels of basic skill achievement differed depending on respective chosen future specialties among residents in Japan. Scores were higher among those pursuing careers in general medical fields and lower among those pursuing highly specialized careers. Residents in training programs devoid of specialty-specific competition may not possess the same motivations as those in competitive systems.

**Supplementary Information:**

The online version contains supplementary material available at 10.1186/s12909-023-04429-4.

## Background

The quality of graduate medical education (GME) training received by resident physicians is challenging to assess [1–3]. It depends on a myriad of factors, including the number and type of clinical experiences, patient population, and the country, geographic region, and hospital site of the training program [3–6]. It also depends on individual residents’ intrinsic motivations, such as time spent acquiring medical knowledge and future career pathway [7]. Standardized examinations assessing knowledge, skills, and attitudes are one of the methods evaluating the GME training programs’ effectiveness, including their ability to equip residents with key clinical skills to provide safe, effective patient care [2, 8]. For example, the United States Medical Licensing Examination (USMLE) has historically been used to assess medical student knowledge and, in some GME programs, determine candidacy for competitive residency positions within different specialties. Indeed, one study demonstrated an association between residency specialty match and scores on the USMLE examinations in the USA [3, 9].

Important differences exist between training systems in different countries. In Japan, the current training program has only been in place since 2004 [10, 11]. After completing a six-year medical school, trainees in Japan are referred to as “residents” and spend the next two post-graduate years acquiring essential clinical skills in one of seven unique specialties (internal medicine, surgery, anesthesiology, pediatrics, psychiatry, obstetrics and gynecology, emergency medicine, and community medicine) and pursuing their future specialty [10, 11]. The purpose of the compulsory post clinical training program as stipulated by the Japanese government is to foster the development of a physician's character and to facilitate the acquisition of fundamental medical skills necessary for adequately addressing prevalent injuries and illnesses encountered in general practice. This is undertaken while acknowledging the social role that medicine and medical care ought to play, irrespective of the specific field they may choose to specialize in the future (Article 16–2, Sect. (1) of the Medical Practitioners Act). Thereafter, three to five additional years are spent training within the chosen field. Unlike in the USA, residents in the Japanese training system may choose their career pathway without specific external competition or need for a certain level of achievement on standardized examinations [7, 10, 11].

In addition to defining individual trainee progress, standardized examinations may provide critical feedback to programs that are participating in nascent training systems like the one in Japan, so that they may identify issues within the education system or allow adjustment of educational strategies [6, 12–14]. One such examination, the internationally validated General Medicine In-Training Examination (GM-ITE), has been incorporated as an annual assessment into many Japanese training centers [8]. The GM-ITE is the Essential Clinical Skills Assessment Test, aimed to facilitate an objective appraisal of the residents' comprehensive clinical competencies as mandated by the Japanese Ministry of Health, Labour and Welfare (MHLW), and is utilized with the intention of pinpointing focal areas and formulating training programs to garner general clinical skills, as well as for the assessment and enhancement of training programs at each medical institution. The Japan Association for the Advancement of Medical Education (JAMEP), a non-profit organization with experienced physicians and peer reviewers, administers the examination.

Prior studies has shown that GM-ITE scores are higher for residents training in rural settings, [6] those with more rotations in general medicine departments, [4] and those with at least a moderate or higher number of patients at any given time [15–17]. Given that residents in Japan may choose their future specialty without external competition (i.e., certain examination score attainment for specific specialties), [10, 18] their intrinsic motivations and perhaps the pursuing specialty may influence their respective levels of knowledge and skill acquisition much more than residents in other countries. However, it is unclear if there exists an association between clinical proficiency (as assessed by the GM-ITE) and their pursuing specialty.

This study’s primary objective was to examine the relative achievement of fundamental skills assessed by the GM-ITE based on pursuing career specialty among residents in the Japanese training system. The secondary objective was to explore hospital and training program characteristics as well as resident factors (e.g., work type and amount, study habits) that may contribute to proficiency in the GM-ITE using the lens of future career specialty.

## Methods

### Participants

**Fig. 1:**
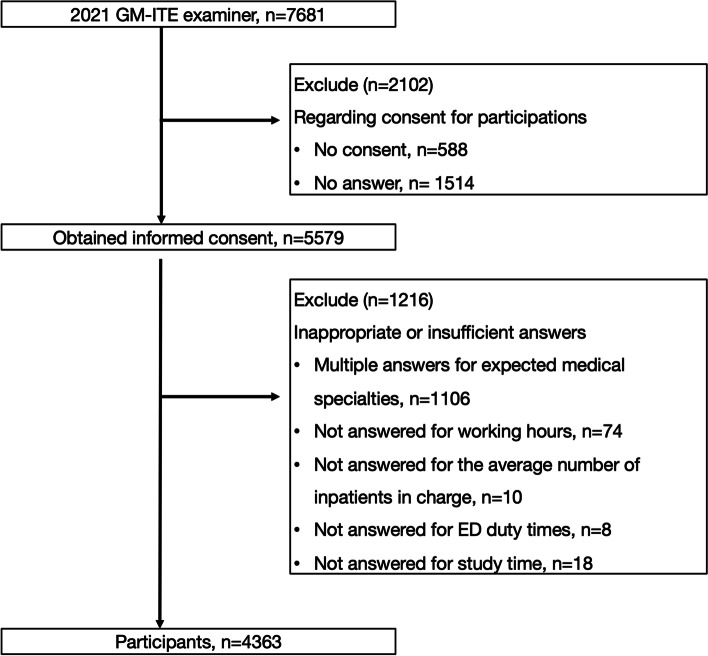
Flow chart of study participants. *Note:* First (PGY-1) and second (PGY-2) year residents who had completed the GM-ITE were surveyed between January 18, 2021 and March 31, 2021. The residents were included if they provided informed consent and completed the electronic survey

This is a nationwide cross-sectional study of medical residents in Japan. First-year (PGY-1) and second-year (PGY-2) residents took the same GM-ITE exam, in which study participants answered the surveys consecutively immediately following the exam. The study was conducted between January 18, 2021 and March 31, 2021. This study followed the STROBE guidelines. Residents were included if they had just completed the GM-ITE, provided informed consent, and completed the electronic survey (Fig. 1). Residents were excluded if they did not respond to the survey (*n* = 1,514), did not provide consent (*n* = 588), indicated more than one preferred specialty (*n* = 1,106), or did not respond to specific survey questions, including the average number of hours worked (*n* = 74), the average number of patients (*n* = 10), emergency department shift amounts (*n* = 8), and time spent studying (*n* = 18).

### Main measures

The GM-ITE assesses general clinical knowledge and its application according to the core curriculum of the training programs of the Ministry of Health, Labour and Welfare (MHLW) [19]. It evaluates individual residents, residency programs, and the clinical training system [8]. The GM-ITE comprises 80 multiple-choice questions and is completed by over 50% of all Japanese medical residents every year (sample GM-ITE questions translated into English are shown in Supplement 1) [8]. In line with the Japanese MHLW’s goals for residents, the 2021 GM-ITE consisted of four domains of basic clinical knowledge: 1) medical interview and professionalism, 2) symptomatology and clinical reasoning, 3) physical examination and treatment, and 4) detailed disease knowledge. The examination assesses the most frequent skills in various fields acquired during initial training. Upon completion of the examination, residents receive feedback based on their relative scores and detailed explanations for each question. Our primary outcome was the total score and the scores in each domain described above.

### Data collection

Immediately following the GM-ITE examination, resident participants provided consent and completed an electronic survey that assessed their work and educational environment, future pursuing specialty, and sociodemographic details. We also surveyed duration of internal medicine rotation, number of emergency department shifts per month which included both walk-in patient settings and cases where patients were transported by ambulance, average daily number of inpatients for whom they provided care, average resident duty hours worked per week, and average time spent studying per week. Characteristic information on each hospital was obtained from the Resident Electronic Information System website [20] and the Foundation for the Advancement of Medical Training [7].

### Statistical analysis

We used the above GM-ITE total score and scores on four domains as the primary outcome (independent variable). All analyses were performed using Stata statistical software (Stata Corp. 2015, Stata 17 Base Reference Manual). Standard descriptive statistics were used to calculate each data set’s number, proportion, mean, median, and interquartile range (IQR). The chi-square, or Fisher’s exact test, was used to compare categorical data. Additionally, multivariate linear regression and multivariate logistic regression analyses were performed to examine factors associated with scores. Department-specific scores were adjusted based on clinical relevance and prior studies [4, 6, 8, 15–18, 21–24]. Furthermore, we assessed the multicollinearity of the multivariate model employing Variance Inflation Factor (VIF) to scrutinize the influence of the aforementioned variables on the GM-ITE score. Dunnett's post hoc tests run after a significant one-way analysis of variance (ANOVA), to determine which differences are significant. Finally, a sensitivity analysis was performed, incorporating several factors into a multivariate regression analysis. All tests were two-tailed, and the statistical significance was set at *p* < 0.05.

## Results

A total of 4,363 residents were included in the analysis. Table 1 shows hospital- and resident-level variables as well as GM-ITE score details as they pertain to residents’ pursuing future specialty. Internal medicine garnered the most interest (*n* = 1,433, 32.8%), followed by surgery (*n* = 408, 9.4%), orthopedics (*n* = 305, 7.0%), pediatrics (*n* = 267, 6.1%), and obstetrics and gynecology (*n* = 214, 4.9%). Community hospitals accounted for 81.3%, hospitals in rural cities accounted for 67.7%, and hospitals overall contained an average of 559 beds. Among all participants, 69.0% were male, and 58.7% were PGY-2 residents. The largest group of residents (71.0%) had 3–5 shifts per month in the emergency department, and 9.1% had 6 or more. The most common number of patients assigned to a resident was 5–9 (55.7%), followed by 0–4 (29.6%) and 10–14 (9.2%). Residents worked an average of fewer than 59 h per week (40.5%), 60–79 h (35.6%), and more than 80 h (24.0%). Finally, 40.2% studied for less than 30 min per week, 39.7% for 31–60 min, 12.8% for 61–90 min, and 3.6% for 91 min or more.

**Table 1:** Background factors and residents’ characteristics, among future specialty

	ALL	Internal Medicine	Surgery	Pediatrics	Obstetrics & Gynecology	Psychiatry	Dermatology	Ophthalmology	Otorhinolary-ngology	Urology	Orthopedics
	4363	1433 (32.84)	408 (9.35)	267 (6.12)	214 (4.90)	173 (3.97)	109 (2.50)	118 (2.70)	96 (2.20)	121 (2.77)	305 (6.99)
Hospital-level variables (%)											
Hospital type											
University	489 (11.21)	153 (10.68)	31 (7.60)	22 (8.24)	28 (13.08)	34 (19.65)	22 (20.18)	21 (17.80)	7 (7.29)	8 (6.61)	28 (9.18)
University branch	325 (7.45)	115 (8.03)	31 (7.60)	22 (8.24)	16 (7.48)	8 (4.62)	12 (11.01)	15 (12.71)	10 (10.42)	6 (4.96)	22 (7.21)
Community	3549 (81.34)	1165 (81.3)	346 (84.80)	223 (83.52)	170 (79.44)	131 (75.72)	75 (68.81)	82 (69.49)	79 (82.29)	107 (88.43)	255 (83.61)
Hospital location											
Urban	1408 (32.27)	515 (35.94)	135 (33.09)	91 (34.08)	75 (35.05)	47 (27.17)	36 (33.03)	40 (33.90)	25 (26.04)	35 (28.93)	92 (30.16)
Rural	2955 (67.73)	918 (64.06)	273 (66.91)	176 (65.92)	139 (64.95)	126 (72.83)	73 (66.97)	78 (66.10)	71 (73.96)	86 (71.07)	213 (69.84)
Number of beds	558.74 (228.99)	564.87 (227.77)	570.67 (229.35)	586.87 (232.57)	608.24 (226.2)	529.53 (239.82)	593.55 (254.93)	549.13 (242.36)	549.6 (198.19)	548.4 (204.13)	537.65 (220.65)
Resident-level variables (%)											
Sex											
Men	3009 (68.97)	1026 (71.60)	307 (75.25)	156 (58.43)	71 (33.18)	132 (76.30)	51 (46.79)	69 (58.47)	58 (60.42)	104 (85.95)	277 (90.82)
Women	1354 (31.03)	407 (28.40)	101 (24.75)	111 (41.57)	143 (66.82)	41 (23.70)	58 (53.21)	49 (41.53)	38 (39.58)	17 (14.05)	28 (9.18)
Post-graduate training level											
PGY-1	1801 (41.28)	615 (42.92)	149 (36.52)	104 (38.95)	74 (34.58)	51 (29.48)	32 (29.36)	44 (37.29)	29 (30.21)	37 (30.58)	115 (37.7)
PGY-2	2562 (58.72)	818 (57.08)	259 (63.48)	163 (61.05)	140 (65.42)	122 (70.52)	77 (70.64)	74 (62.71)	67 (69.79)	84 (69.42)	190 (62.3)
Emergency department shifts per month											
None	152 (3.48)	44 (3.07)	9 (2.21)	9 (3.37)	8 (3.74)	8 (4.62)	5 (4.59)	6 (5.08)	6 (6.25)		9 (2.95)
1–2	688 (15.77)	217 (15.14)	57 (13.97)	38 (14.23)	34 (15.89)	39 (22.54)	33 (30.28)	23 (19.49)	15 (15.63)	19 (15.7)	33 (10.82)
3–5	3099 (71.03)	1051 (73.34)	290 (71.08)	194 (72.66)	157 (73.36)	115 (66.47)	62 (56.88)	81 (68.64)	68 (70.83)	87 (71.9)	222 (72.79)
6 or more	396 (9.08)	115 (8.03)	50 (12.25)	26 (9.74)	12 (5.61)	10 (5.78)	7 (6.42)	7 (5.93)	6 (6.25)	14 (11.57)	40 (13.11)
Unknown	28 (0.64)	6 (0.42)	2 (0.49)		3 (1.40)	1 (0.58)	2 (1.83)	1 (0.85)	1 (1.04)	1 (0.83)	1 (0.33)
Average number of inpatients assigned											
0–4	1292 (29.61)	365 (25.47)	111 (27.21)	79 (29.59)	68 (31.78)	66 (38.15)	34 (31.19)	41 (34.75)	43 (44.79)	44 (36.36)	92 (30.16)
5–9	2428 (55.65)	859 (59.94)	225 (55.15)	152 (56.93)	123 (57.48)	78 (45.09)	60 (55.05)	58 (49.15)	44 (45.83)	62 (51.24)	168 (55.08)
10–14	402 (9.21)	146 (10.19)	48 (11.76)	21 (7.87)	13 (6.07)	22 (12.72)	12 (11.01)	14 (11.86)	5 (5.21)	8 (6.61)	26 (8.52)
15 or more	115 (2.64)	35 (2.44)	12 (2.94)	6 (2.25)	4 (1.87)	3 (1.73)		3 (2.54)	3 (3.13)	2 (1.65)	7 (2.30)
Unknown	126 (2.89)	28 (1.95)	12 (2.94)	9 (3.37)	6 (2.80)	4 (2.31)	3 (2.75)	2 (1.69)	1 (1.04)	5 (4.13)	12 (3.93)
Average duty hours worked per week											
59 or fewer	1765 (40.45)	554 (38.66)	142 (34.80)	108 (40.45)	77 (35.98)	98 (56.65)	52 (47.71)	62 (52.54)	37 (38.54)	50 (41.32)	107 (35.08)
60–79	1552 (35.57)	515 (35.94)	142 (34.80)	98 (36.7)	83 (38.79)	62 (35.84)	31 (28.44)	34 (28.81)	37 (38.54)	44 (36.36)	108 (35.41)
80 or more	1046 (23.97)	364 (25.40)	124 (30.39)	61 (22.85)	54 (25.23)	13 (7.51)	26 (23.85)	22 (18.64)	22 (22.92)	27 (22.31)	90 (29.51)
Average time spent studying per week											
0–30 min	1752 (40.16)	513 (35.80)	152 (37.25)	100 (37.45)	87 (40.65)	90 (52.02)	55 (50.46)	54 (45.76)	38 (39.58)	56 (46.28)	134 (43.93)
31–60 min	1734 (39.74)	583 (40.68)	164 (40.20)	119 (44.57)	101 (47.20)	55 (31.79)	34 (31.19)	45 (38.14)	45 (46.88)	49 (40.5)	105 (34.43)
61–90 min	560 (12.84)	225 (15.70)	63 (15.44)	33 (12.36)	18 (8.41)	18 (10.40)	11 (10.09)	12 (10.17)	8 (8.33)	12 (9.92)	43 (14.10)
91 min or more	158 (3.62)	71 (4.95)	15 (3.68)	10 (3.75)	3 (1.40)	3 (1.73)	2 (1.83)	2 (1.69)	2 (2.08)	1 (0.83)	8 (2.62)
None	159 (3.64)	41 (2.86)	14 (3.43)	5 (1.87)	5 (2.34)	7 (4.05)	7 (6.42)	5 (4.24)	3 (3.13)	3 (2.48)	15 (4.92)
GM-ITE score (SD)		20.65	19.04			56.07	56.88	50			
Total; Max 80	44.62 (6.94)	45.86 (7.01)	44.69 (6.44)	44.74 (6.37)	45.39 (5.95)	43.34 (6.79)	42.06 (6.23)	40.44 (5.91)	42.72 (6.41)	43.77 (6.79)	42.75 (6.3)
Medical interview and professionalism; Max 8	6.26 (1.11)	6.34 (1.06)	6.15 (1.11)	6.33 (1.15)	6.27 (1.06)	6.34 (1.14)	6.19 (1.08)	6.07 (1.17)	6.23 (1.25)	6.2 (1.12)	6.09 (1.14)
Symptomatology and clinical reasoning; Max 18	10.92 (2.35)	11.39 (2.38)	11.09 (2.19)	10.74 (2.17)	11.01 (2.2)	10.45 (2.17)	9.94 (2.21)	9.71 (2.05)	10.2 (2.25)	10.95 (2.29)	10.3 (2.24)
Physical examination and procedure; Max 18	9.42 (2.22)	9.68 (2.28)	9.33 (2.09)	9.4 (2.09)	9.76 (2.26)	8.97 (2)	8.77 (2.23)	8.64 (2.06)	9.09 (1.95)	9.04 (2.05)	8.97 (2.09)
Disease knowledge; Max 36	18.02 (3.92)	18.44 (3.95)	18.12 (3.78)	18.27 (3.85)	18.35 (3.54)	17.58 (4.2)	17.16 (3.45)	16.03 (3.98)	17.2 (3.71)	17.58 (4.01)	17.41 (3.57)

	Neurosurgery	Plastic Surgery	Emergency Medicine	Anesthesiology	Radiology	Rehabilitation Medicine	Pathology	Clinical Laboratory	General Medicine	Other	Not decided
	117 (2.68)	83 (1.9)	126 (2.89)	165 (3.78)	93 (2.13)	31 (0.71)	27 (0.62)	3 (0.07)	101 (2.31)	76 (1.74)	297 (6.81)
Hospital-level variables (%)											
Hospital type											
University	9 (7.69)	7 (8.43)	19 (15.08)	17 (10.3)	21 (22.58)	6 (19.35)	4 (14.81)	1 (33.33)	14 (13.86)	6 (7.89)	31 (10.44)
University branch	2 (1.71)	7 (8.43)	7 (5.56)	13 (7.88)	3 (3.23)	1 (3.23)	1 (3.70)		6 (5.94)	3 (3.95)	25 (8.42)
Community	106 (90.60)	69 (83.13)	100 (79.37)	135 (81.82)	69 (74.19)	24 (77.42)	22 (81.50)	2 (66.67)	81 (80.20)	67 (88.16)	241 (81.14)
Hospital location											
Urban	28 (23.93)	33 (39.76)	33 (26.19)	52 (31.52)	25 (26.88)	10 (32.26)	14 (51.85)	1 (33.33)	22 (21.78)	25 (32.89)	74 (24.92)
Rural	89 (76.07)	50 (60.24)	93 (73.81)	113 (68.48)	68 (73.12)	21 (67.74)	13 (48.15)	2 (66.67)	79 (78.22)	51 (67.11)	223 (75.08)
Number of beds	529.03 (216.92)	534.31 (235.37)	605.04 (251.44)	552.89 (219.34)	566.95 (212.39)	513.61 (283.45)	497.48 (198.3)	523.33 (430.85)	479.3 (231.05)	526.91 (234.78)	534.1 (220.61)
Resident-level variables (%)											
Sex											
Men	102 (87.18)	45 (54.22)	89 (70.63)	91 (55.15)	79 (84.95)	19 (61.29)	19 (70.37)	2 (66.67)	69 (68.32)	54 (71.05)	189 (63.64)
Women	15 (12.82)	38 (45.78)	37 (29.37)	74 (44.85)	14 (15.05)	12 (38.71)	8 (29.63)	1 (33.33)	32 (31.68)	22 (28.95)	108 (36.36)
Post-graduate training level											
PGY-1	49 (41.88)	24 (28.92)	38 (30.16)	50 (30.3)	28 (30.11)	13 (41.94)	12 (44.44)	3 (100)	33 (32.67)	22 (28.95)	279 (93.94)
PGY-2	68 (58.12)	59 (71.08)	88 (69.84)	115 (69.7)	65 (69.89)	18 (58.06)	15 (55.56)		68 (67.33)	54 (71.05)	18 (6.06)
Emergency department shifts per month											
None	1 (0.85)	2 (2.41)	5 (3.97)	8 (4.85)	5 (5.38)			1 (33.33)	3 (2.97)	6 (7.89)	17 (5.72)
1–2	11 (9.40)	13 (15.66)	21 (16.67)	25 (15.15)	17 (18.28)	6 (19.35)	8 (29.63)		18 (17.82)	17 (22.37)	44 (14.81)
3–5	89 (76.07)	60 (72.29)	86 (68.25)	114 (69.09)	64 (68.82)	23 (74.19)	17 (62.96)	2 (66.67)	71 (70.30)	45 (59.21)	201 (67.68)
6 or more	16 (13.68)	8 (9.64)	13 (10.32)	17 (10.30)	5 (5.38)	1 (3.23)	2 (7.41)		8 (7.92)	8 (10.53)	31 (10.44)
Unknown			1 (0.79)	1 (0.61)	2 (2.15)	1 (3.23)			1 (0.99)		4 (1.35)
Average number of inpatients assigned											
0–4	29 (24.79)	28 (33.73)	34 (26.98)	37 (22.42)	44 (47.31)	12 (38.71)	14 (51.85)	1 (33.33)	31 (30.69)	25 (32.89)	94 (31.65)
5–9	57 (48.72)	45 (54.22)	76 (60.32)	98 (59.39)	42 (45.16)	15 (48.39)	12 (44.44)	2 (66.67)	53 (52.48)	41 (53.95)	158 (53.2)
10–14	16 (13.68)	8 (9.64)	11 (8.73)	13 (7.88)	3 (3.23)	2 (6.45)	1 (3.70)		9 (8.91)	4 (5.26)	20 (6.73)
15 or more	10 (8.55)		5 (3.97)	6 (3.64)	1 (1.08)				5 (4.95)	3 (3.95)	10 (3.37)
Unknown	5 (4.27)	2 (2.41)		11 (6.67)	3 (3.23)	2 (6.45)			3 (2.97)	3 (3.95)	15 (5.05)
Average duty hours worked per week											
59 or fewer	33 (28.21)	30 (36.14)	54 (42.86)	70 (42.42)	51 (54.84)	14 (45.16)	20 (74.07)	2 (66.67)	38 (37.62)	38 (50.00)	128 (43.1)
60–79	42 (35.9)	40 (48.19)	45 (35.71)	62 (37.58)	27 (29.03)	8 (25.81)	5 (18.52)	1 (33.33)	45 (44.55)	21 (27.63)	102 (34.34)
80 or more	42 (35.9)	13 (15.66)	27 (21.43)	33 (20)	15 (16.13)	9 (29.03)	2 (7.41)		18 (17.82)	17 (22.37)	67 (22.56)
Average time spent studying per week											
0–30 min	39 (33.33)	37 (44.58)	49 (38.89)	69 (41.82)	36 (38.71)	11 (35.48)	7 (25.93)	1 (33.33)	32 (31.68)	42 (55.26)	150 (50.51)
31–60 min	47 (40.17)	31 (37.35)	52 (41.27)	75 (45.45)	40 (43.01)	15 (48.39)	11 (40.74)	1 (33.33)	42 (41.58)	22 (28.95)	98 (33.0)
61–90 min	18 (15.38)	9 (10.84)	15 (11.9)	11 (6.67)	6 (6.45)		6 (22.22)	1 (33.33)	20 (19.80)	3 (3.95)	28 (9.43)
91 min or more	10 (8.55)	1 (1.20)	6 (4.76)	5 (3.03)	7 (7.53)		2 (7.41)		5 (4.95)	2 (2.63)	3 (1.01)
None	3 (2.56)	5 (6.02)	4 (3.17)	5 (3.03)	4 (4.3)	5 (16.13)	1 (3.7)		2 (1.98)	7 (9.21)	18 (6.06)
GM-ITE score (SD)	23.93					51.61			24.75	64.47	56.57
Total; Max 80	45.74 (7.68)	42.31 (6.95)	46.98 (7.56)	44.75 (6.63)	44.6 (6.84)	41.84 (6.89)	44.59 (7.12)	45 (6.24)	47.66 (7.08)	42.62 (7.42)	43.05 (7.09)
Medical interview and professionalism; Max 8	6.22 (1.17)	6.22 (1.02)	6.29 (1.05)	6.18 (1.19)	6.27 (1.19)	6.29 (1.22)	6.63 (1.01)	7.67 (0.58)	6.51 (1.04)	6.07 (1.39)	6.21 (1.13)
Symptomatology and clinical reasoning; Max 18	11.28 (2.51)	10.19 (2.3)	11.63 (2.43)	10.58 (2.2)	10.86 (2.32)	9.87 (2.09)	10.7 (2.38)	9.67 (2.89)	11.83 (2.28)	10.21 (2.43)	10.48 (2.26)
Physical examination and procedure; Max 18	9.67 (2.18)	9.29 (2.18)	10 (2.4)	9.37 (2.28)	9.59 (2.02)	8.68 (1.97)	9.41 (1.91)	9 (4.58)	9.98 (2.24)	9.22 (2.18)	9.16 (2.27)
Disease knowledge; Max 36	18.57 (4.45)	16.61 (4.02)	19.07 (3.78)	18.61 (3.54)	17.88 (3.73)	17 (4.35)	17.85 (3.9)	18.67 (3.06)	19.34 (3.84)	17.12 (4.1)	17.19 (3.97)

*Abbreviations PGY-1* First year residents, *PGY-2* Second year residents, *ED* Emergency Department, *GM-ITE* General Medicine In-Training Examination, *SD* Standard Deviation

Table 2 presents the mean of the total scores and standard deviations of GM-ITE pertaining to each hospital-level, resident-level, and their pursuing future specialty variables. The average score demonstrated an upward trend proportional to the frequency of Emergency Department duties per month, and a similar positive correlation was observed with the average score as the weekly study hours escalated. Nevertheless, no substantial disparities were discernible in terms of gender, Postgraduate Year, or urban residency. Furthermore, Table 2 also shows the correlations between the GM-ITE total scores and the multivariate linear analysis of the future pursuing specialty and individual factors. The VIF estimations for all predictive variables were ascertained to reside within the permissible spectrum in our multivariate regression analysis (mean VIF = 1.42, all variables were less than 10). Residents training in community hospitals scored higher than those in university hospitals (coefficient 2.44, 95% CI 1.08 to 3.81; *p* < 0.0001), and there was a positive correlation for total scores as the number of beds increased. There was no difference in scores between residents training in urban hospitals and those training in rural hospitals. There was no difference in scores between male and female residents, but PGY-2 residents scored higher than PGY-1 residents (coefficient 0.90, 95% CI 0.49 to 1.32; *p* < 0.0001). When compared to the referent of zero shifts, there was no significant difference in scores between the number of emergency department (ED) shifts per month. When compared to the referent of 0–4 patients, the average number of patients assigned significantly correlated with higher scores for those assigned 5–9 patients (coefficient 0.80, 95% CI 0.33 to 1.27; *p* < 0.001) and 10–14 patients (coefficient 0.86, 95% CI 0.04 to 1.68; *p* = 0.039), but not for 15 or more patients or unknown. When compared to the referent of 59 or fewer hours, the average amount of resident duty hours worked per week significantly correlated with higher scores for those who worked 60–79 h (coefficient 1.18, 95% CI 0.73 to 1.64; *p* < 0.001) and 80 or more hours (coefficient 0.62, 95% CI 0.10 to 1.15; *p* = 0.020). When compared to the referent of 0–30 min, those residents who spent more than 30 min per week studying tended to have significantly higher scores. Internal medicine was chosen as the referent since it was the most commonly chosen future specialty. When compared to internal medicine, only those residents who chose general medicine achieved higher scores (coefficient 1.38, 95% CI 0.08 to 2.68, *p* = 0.038). Conversely, the nine specialties and “Other/Not decided” groups had significantly lower scores. Moreover, both the PGY-1 and the PGY-2 were segregated into their respective categories, with the distinctive characteristics of each being elucidated utilizing the identical methodology as depicted in Tables 1 and 2. The outcomes exhibited a semblance to the comprehensive table, with scores for both General Medicine and Emergency Medicine exhibiting a propensity towards higher values across both academic years. (Supplementary table 1–3.)

**Table 2:** The mean of the GM-ITE score among variables and the Multivariate Linear Regression Analysis

			95% CI		
	Mean (SD)	Co-efficient	Lower	Upper	p-value
*Hospital-level variables*					
Hospital types					
University	42.03 (6.56)	Reference	-	-	-
University branch	41.91 (5.99)	-0.04	-1.91	1.84	0.969
Community	45.23 (6.93)	2.44	1.08	3.81	< .001
Hospital location					
Urban	44.97 (7.20)	Reference	-	-	-
Rural	44.46 (6.80)	-0.36	-1.01	0.29	0.274
Number of beds (per 100 beds increase)	-	0.21	0.04	0.37	0.013
*Resident-level variables*					
Sex					
Men	44.67 (7.19)	Reference	-	-	-
Women	44.51 (6.33)	-0.01	-0.45	0.42	0.962
PGY					
PGY-1	44.08 (6.76)	Reference			
PGY-2	45.01 (7.03)	0.9	0.49	1.32	< .001
ED duty per month					
None	43.09 (6.55)	Reference	-	-	-
1–2	43.12 (6.63)	-0.58	-1.76	0.59	0.331
3–5	44.91 (6.85)	-0.07	-1.23	1.09	0.911
6 or more	45.71 (7.76)	0.37	-1	1.74	0.598
Unknown	42.29 (6.67)	-0.43	-3	2.15	0.745
Average number of inpatients in charge					
0–4	43.87 (6.59)	Reference	-	-	-
5–9	45.05 (7.06)	0.8	0.33	1.27	< .001
10–14	44.69 (6.90)	0.86	0.04	1.68	0.039
15 or more	44.60 (7.96)	0.32	-1.03	1.68	0.638
Unknown	43.96 (6.37)	-0.11	-1.33	1.11	0.865
Resident duty hour per week					
59 or less	43.68 (6.80)	Reference	-	-	-
60–79	45.42 (6.91)	1.18	0.73	1.64	< .001
80 or more	45.03 (7.03)	0.62	0.1	1.15	0.02
Study hour per week					
0–30 min	43.82 (6.57)	Reference	-	-	-
31–60 min	44.90 (6.88)	0.52	0.09	0.95	0.018
61–90 min	46.15 (7.24)	1.18	0.55	1.8	< .001
91 min or more	46.81 (8.15)	1.26	0.18	2.33	0.022
None	42.79 (7.39)	-0.32	-1.36	0.72	0.548
Specialty					
General Medicine (*n* = 101)	47.66 (7.08)	1.38	0.08	2.68	0.038
Emergency Medicine (*n* = 126)	46.98 (7.56)	1.02	-0.14	2.19	0.084
Clinical Laboratory (*n* = 3)	45.00 (6.24)	0.82	-6.38	8.03	0.823
Internal Medicine (*n* = 1433)	45.86 (7.01)	Reference	-	-	-
Pathology (*n* = 27)	44.59 (7.12)	-0.42	-2.86	2.03	0.739
Radiology (*n* = 93)	44.6 (6.84)	-0.49	-1.84	0.85	0.47
Neurosurgery (*n* = 117)	45.74 (7.68)	-0.57	-1.78	0.64	0.356
Obstetrics and Gynecology (*n* = 214)	45.39 (5.95)	-0.7	-1.63	0.23	0.141
Anesthesiology (*n* = 165)	44.75 (6.63)	-0.99	-2.02	0.04	0.06
Surgery (*n* = 408)	44.69 (6.44)	-1.18	-1.88	-0.48	< .001
Pediatrics (*n* = 267)	44.74 (6.37)	-1.29	-2.12	-0.45	0.003
Psychiatry (*n* = 173)	43.34 (6.79)	-1.76	-2.78	-0.74	< .001
Urology (*n* = 121)	43.77 (6.79)	-1.94	-3.13	-0.75	< .001
Rehabilitation Medicine (*n = *31)	41.84 (6.89)	-2.29	-4.59	0.01	0.051
Dermatology (*n* = 109)	42.06 (6.23)	-2.72	-3.97	-1.47	< .001
Otorhinolaryngology (*n* = 96)	42.72 (6.41)	-2.91	-4.23	-1.59	< .001
Orthopedics (*n* = 305)	42.75 (6.3)	-2.98	-3.78	-2.19	< .001
Plastic Surgery (*n* = 83)	42.31 (6.95)	-3.66	-5.09	-2.23	< .001
Ophthalmology (*n* = 118)	40.44 (5.91)	-4.37	-5.58	-3.17	< .001
Not decided (*n* = 297)	43.05 (7.09)	-1.81	-2.64	-0.98	< .001
Other (*n* = 76)	42.62 (7.42)	-2.38	-3.86	-0.9	0.002

*Abbreviations PGY-1* First year residents, *PGY-2* Second year residents, *GM-ITE* General Medicine In-Training Examination, *CI* Confidence Interval, *ED* Emergency Department

To adjust for potential confounders of clinically significant associated factors for the GM-ITE score, the following variables were incorporated in the multivariate analysis: chosen specialty, hospital type, hospital location, sex, number of post-graduate years, number of emergency department shifts per month, average daily number of inpatients for whom the resident provided care, average resident duty hours worked per week, and average time spent studying per week. When internal medicine (the future specialty chosen by the highest number of residents) was set as the reference, residents planning on choosing general medicine were the only ones who scored higher (coefficient 1.3792, 95% CI 0.07894–2.6796, *p* = 0.0376)

**Fig. 2:**
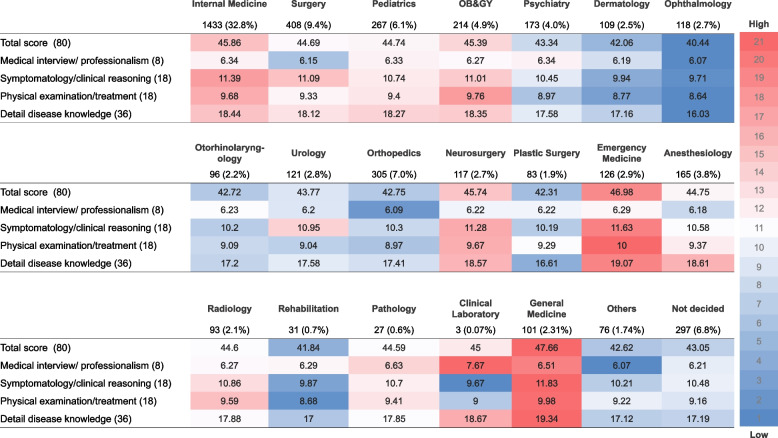
Ranking heatmap of GM-ITE scores by chosen specialty. *Note:* A heat map of the GM-ITE total scores and scores in the four domains, organized according to the residents' chosen specialties, is presented. The colors ranging from red to blue on the rightmost side serve as indicators of the rank order of the scores, which are classified into 21 levels. The left side provides a detailed breakdown of the scores, with the overall score equating to 80 points (1 point per clinical question). It is further divided into medical interview/professionalism (8 points), symptomatology/clinical reasoning (18 points), physical examination/treatment (18 points), and detailed disease knowledge (36 points). Analysis of the data revealed that the attainment of basic clinical skills was generally higher in the specialties of general medicine, emergency medicine, and internal medicine. However, certain specialties exhibited areas of underperformance

**Fig. 3:**
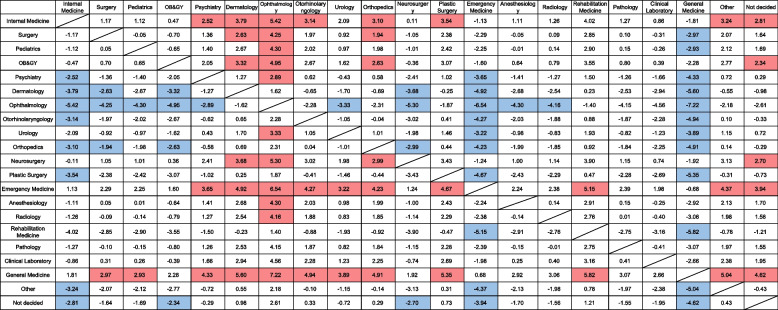
Univariate comparison of GM-ITE scores among medical resident’s pursuing specialty using ANOVA with Dunnett post hoc test. *Note:* For the departments delineated in the left-hand column, the disparity between their scores relative to those departments listed in the corresponding columns is indicated. Highlighted cells denote statistical significance, with an orange hue signifying a positive correlation and blue designating a negative one

In addition, General Medicine scores in PGY-1 demonstrated a statistically significant escalation subsequent to adjustment for Internal Medicine as a benchmark. The residents of PGY-1 manifested a significantly superior performance in General Medicine (coefficient 3.51, 95% Confidence Interval 0.04 to 1.68; *p* = 0.039). Conversely, the disparity between General Medicine and PGY-2 no longer bore statistical significance in the aftermath of the PGY-2 analysis. (Refer to Supplementary table 4 and 5 for additional data). Figure 2 shows a heat map of the GM-ITE total score and scores for its four domains organized by residents’ chosen future specialties. Total scores were higher in general medicine, emergency medicine, and internal medicine, with residents who chose general medicine having higher achievement in basic clinical skills during the study period. Some areas of low performance were also identified among certain future specialties. For example, scores on the medical interview and professionalism tended to be lower in departments involving procedures, such as ophthalmology (6.07/8), orthopedics (6.09/8), surgery (6.15/8), and anesthesia (6.18/8). Scores in symptomatology and clinical reasoning tended to be lower in the clinical laboratory (9.67/18), ophthalmology (9.71/18), and rehabilitation medicine (9.87/18). Similarly, scores for physical examination/treatment were lower among residents whose chosen specialty lacks opportunities to examine the whole body, administer medications, and perform procedures, such as ophthalmology (8.64/18), rehabilitation (8.68/18), and dermatology (8.77/18). Scores for detailed disease knowledge were low in specialties lacking head-to-toe physical examinations, medication treatment, and procedures, such as ophthalmology (16.03/36) and plastic surgery (16.61/36). Next, interesting patterns emerged within individual specialties. In many specialties, all scores aligned (either all or most domain scores were high, as in general medicine, emergency medicine, and internal medicine; or all or most domain scores were low, as in ophthalmology, rehabilitation, dermatology, and orthopedics). However, in other specialties, scores in some domains were higher than in others. For instance, in surgery and anesthesiology, scores for medical interview/professionalism were lower, whereas those for detailed disease knowledge were higher. In urology, symptomatology/clinical reasoning scores were higher, whereas all other domains were lower. Finally, we elucidate the outcomes of Dunnett's test as a post-hoc appraisal to authenticate the divergence in scores corresponding to each medical specialty (Fig. 3). Within this illustration, upon scrutinizing the axis of medical specialties in the leftmost column, it is observed that general medicine, internal medicine, and emergency medicine manifest significantly augmented scores. Conversely, the scores of residents nurturing aspirations to delve into highly specialized domains such as ophthalmology, dermatology, orthopedics, and plastic surgery in the impending future demonstrated a propensity towards lower values in this examination, which gauges essential clinical training proficiency.

## Discussion

In this nationwide, cross-sectional study, we examined associations among clinical proficiency and skill as assessed by the GM-ITE, training program characteristics, individual resident factors, and pursuing career specialty among resident physicians in Japan. We found that scores on the standardized GM-ITE assessment were higher for residents who planned careers in general medicine, emergency medicine, and internal medicine and lower for residents who planned for highly specialized departments that do not offer general practice. We also discovered distinctive trends based on hospital and resident attributes. GM-ITE scores were higher for those who trained in community hospitals with higher numbers of beds, who were more advanced in their training (i.e., PGY-2), and who spent more time working and studying. Higher scores were noted for residents who cared for a moderate but not extreme number of patients at a time.

In Japan, general medicine is a new specialty certification, established recently in 2018 [23, 25]. General medicine training includes care in the outpatient clinic, inpatient ward, and emergency department settings. Moreover, a resident may thereafter choose a career pathway among family medicine, hospital medicine, or general internal medicine [25]. One potential explanation of our data is that Japanese general medicine physicians cover a wide range of settings and contexts; this breadth of experience facilitates the acquisition of basic clinical skills (knowledge, skills, and attitudes) needed for success on the GM-ITE. This is supported by the fact that previous GM-ITE studies have shown higher scores among those who have completed a general medicine rotation [4, 23, 24]. It is not surprising that those residents with more clinical experience (in the form of time spent in practice and exposure to those clinical conditions most likely to be tested on the GM-ITE) fare better on the examination. In other countries also, postgraduate training examinations have shown that extensive comprehensive training results in higher examination scores [3]. It has been noted that learners in Japan who are passionate about lifelong comprehensive and extensive learning tend to go on to become general medicine physicians [6, 17, 23].

Nearly all physicians in Japan choose their specialty in the second postgraduate year. As a comparative example, trainees in the USA often select a residency program in the specialty of their coice before graduating from medical school. This decision may be based partly on USMLE performance, although the transition to pass/fail assessment for USMLE Step 1 has changed this dynamic. Unlike in Japan, considerable examination competition exists in the USA [7, 9, 26]. Competitive fields such as dermatology, otolaryngology, plastic surgery, and ophthalmology generally require high USMLE Step 2 score scores to be considered [9]. In the USA, annual incomes vary by specialty, with significant differences noted [26]. This pay discrepancy may be an external motivating factor in future career, in that departments with high income may attract residents with good performance in consideration of repayment of large student loans [26]. In Japan, however, there is no competition for nor restrictions on pursuing specific medical specialties. Additionally, physician income in Japan does not vary significantly between departments, further lowering competition compared with the system in the USA [7, 9, 18, 26, 27]. In a large-scale survey in Japan, high remuneration was not a contributing factor in matching training hospitals [18]. Instead, junior career Japanese physicians often decide their career pathways based on their medical interests and curiosity, the scope of their practice, ease of work, expected duties, and alignment of work and abilities [11, 18, 27].

Does the training environment influence residents’ future pursuing specialty, or do they choose the training environment based on their defined future career pathway? We cannot explain any potential or real causal relationships. However, the differences in examination scores according to the chosen future specialty may be due to differences in motivation and the actual amount of active learning during the two years of mandatory rotational training although this was not measured in this study [16, 21]. In addition, university hospitals in Japan tend to focus on experimental research achievement [28, 29] and do not provide the same levels of training in primary and general health care as community hospitals [6, 23]. In our study, a large proportion of residents who seek careers in highly specialized fields like ophthalmology and dermatology are trained at university hospitals, where the number of work shifts, amount of time worked, and amount of clinical experience tend to be smaller. Furthermore, it has been shown that residents who work fewer hours have significantly less time for actual independent study, despite having relatively more time overall, [21] in which case less studying correlates with lower GM-ITE scores [16, 21]. Considering all of the above, it is possible that in Japan, highly specialized departments, such as ophthalmology, dermatology, and plastic surgery, are not expected to have a wide range of clinical skills or knowledge related to systemic conditions. Thus, those who wish to pursue these careers may not have the motivation and study time to devote to skills assessed by the GM-ITE.

Our findings must be interpreted in the context of several limitations. First, this is a cross-sectional study, and it is unknown whether PGY-1 residents accurately predict their actual future specialties. In fact, the commencement of the GM-ITE coincides with the culmination of the academic year, suggesting a greater probability that PGY-2 residents would have delineated their career trajectory by this juncture compared to PGY-1. Furthermore, the GM-ITE scores even before the initiation of the PGY-1 residency are unknown, and the contemplation of future assessments to ascertain this before mandatory clinical training commences is currently under investigation. Second, the presence of selection bias is inevitable. Despite the test being taken by more than a half of residents nationwide, we exclude data from respondents who indicated more than one division from this cohort. Inclusion of such data might engender alterations in the results. Third, the history of the general medicine specialty in Japan is young, and there is mutual overlap among the fields of general internal medicine, hospital medicine, and family medicine, all of which are often also responsible for general internal medicine ward and outpatient services [25]. On the other hand, there may be residents in internal medicine who wish to choose hospital medicine or general internal medicine after their residency program. This overlap can be a major misclassification bias [25, 29, 30]. Subsequently, our GM-ITE data exclusively displays the four categories of examination questions and the total score, as articulated above. This may be because certain questions may appraise the attitude towards physical examination and interview, even when the disease is distinctly categorized under obstetrics and gynecology in the medical department classification. Consequently, it was not feasible to demonstrate a correlation between which medical department applicants are more likely to encounter questions in specific medical department categories. Nonetheless, this is congruent with the MHLW's objective of training 'physicians who can provide comprehensive and wide-ranging medical care. Finally, the results of this examination may serve as either intrinsic or extrinsic motivation for resident aspirants. Even though this examination does not exert a direct influence on career progression or retention, the results are communicated to the program director. Consequently, there exists a potential bias, such as residents who harbor future intentions to apply to a broader array of departments, inclusive of general practice or emergency medicine, may approach the examination with heightened diligence and engage in more rigorous preparation.

## Conclusion

In conclusion, this nationwide study is the first of its kind to reveal differing levels of attainment of basic clinical skills, as assessed by the GM-ITE, among residents in Japan according to their chosen specialty. In particular, higher scores were noted among those pursuing careers in general medicine and emergency medicine fields and lower scores among those pursuing highly subspecialized careers. Differences were also observed among future departments with respect to the amount of time spent caring for patients and studying and the number of assigned patients. Residents in medical training programs devoid of specialty-specific competition may not possess the same motivations as competitive systems, and countries may learn from these experiences. Further research is needed to address potential causative factors for these disparate levels of achievement and associations, including perhaps intrinsic motivations to learn within fields outside of the chosen career pathway during the two years of required training. Future studies should also seek to elucidate strategies to improve clinical skills for residents with low achievement levels.

## Supplementary Information

**Additional file 1.** Background factors and PGY-1 residents’ characteristics, among each pursuing specialty.

**Additional file 2.** Background factors and PGY-2 residents’ characteristics, among each pursuing specialty.

**Additional file 3.** Mean scores with standard deviation of GM-ITE among each pursuing specialty for PGY-1 and PGY2.

**Additional file 4.** The Multivariate Linear Regression Analysis of GM-ITE score for PGY-1.

**Additional file 5.** The Multivariate Linear Regression Analysis of GM-ITE score for PGY-2.

**Additional file 6: Online-Only Supplements 1.** Sample questions from the GM-ITE examination translated into the English language.

## Data Availability

The data that support the findings of this study are available from the corresponding author, T.W, the General Medicine Center, Shimane University Hospital (E-mail. wataritari@gmail.com), upon reasonable request.

## Abbreviations

GM-ITE: General Medicine In-Training Examination
GME: Graduate medical education
USMLE: United States Medical Licensing Examination
MHLW: Ministry of Health, Labour and Welfare
PGY-1: First year resident
PGY-2: Second year resident
JAMEP: Japan Association for the Advancement of Medical Education
IQR: Interquartile range
