# Supplementary material for: Medical resident’s pursuing specialty and differences in clinical proficiency among medical residents in Japan: a nationwide cross-sectional study
Source: BMC Med Educ. 2023 Jun 22;23:464. doi: 10.1186/s12909-023-04429-4 (PMC10286340; doi:10.1186/s12909-023-04429-4)
Supplement: Supplementary file 6 — Additional file 6: Online-Only Supplements 1. Sample questions from the GM-ITE examination translated into the English language. [file 12909_2023_4429_MOESM6_ESM.docx]

**Online-Only Supplements. 1** Sample questions from the GM-ITE examination translated into the English language

**Physical Examination and Clinical Procedures**

A previously healthy 46-year-old man is seeking care for twitching in his left arm. Neurological and other physical findings are normal.

A video showing the findings in the left upper arm is available at the following link.

What disease do these findings suggest?


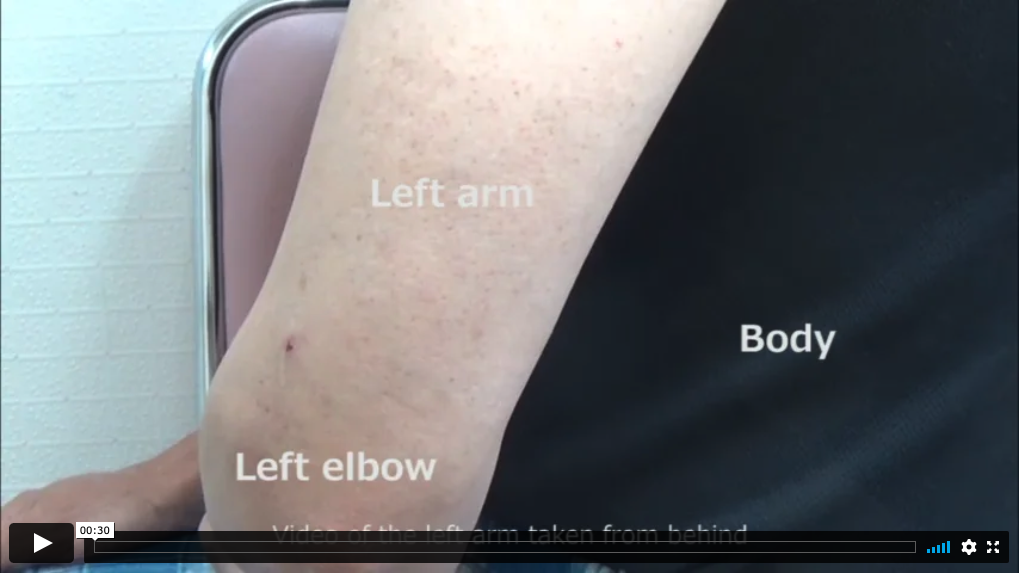


| 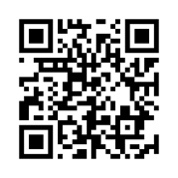 | https://vimeo.com/488752675/6fd2ad2f8a |
| --- | --- |

(1)　Amyotrophic lateral sclerosis (ALS)

(2)　Antiphospholipid syndrome

(3)　Creutzfeldt-Jakob disease

(4)　Parkinson’s disease

(5)　Lance-Adams syndrome

**Answer**

(1)　Amyotrophic lateral sclerosis (ALS)

**Commentary**

When examining a patient with involuntary movements, clinicians observing the phenomenon must describe it as exactly as it appears and must also be acquainted with generally known symptoms.

Of the attempted approaches to classification, an approach that classifies movement according to the site involved is relatively easy to grasp. It focuses on (1) movement at the level of the muscle fiber and/or bundle, (2) movement extending to one or several muscles, and (3) movement at the level affecting the limbs and/or trunk. The involuntary contractions seen in the video, which seem to occur in a single muscle and feature no joint movement, are classifiable as “(1) movement at the level of the muscle bundle.” This category includes fasciculation and myokymia.

This type of involuntary movement signifies diseases involving lower motor neurons, such as amyotrophic lateral sclerosis (ALS), multifocal motor neuropathy, radiation plexopathy, and Isaacs’ syndrome. In this case, myokymia was electromyographically confirmed. Myokymia is a relatively rare electromyographic anomaly in ALS but does appear in clinical practice. Choice 1 is, therefore, correct.

Antiphospholipid syndrome frequently causes chorea, and Creutzfeldt-Jakob disease is characterized by myoclonus. Although Parkinson’s disease features a variety of involuntary movements, resting tremors are most typical. Lance-Adams syndrome, which follows hypoxic encephalopathy, is an action myoclonus caused by cortical excitation.

**References**

1. Whaley NR, Rubin DI. Myokimic discharges in amyotrophic lateral sclerosis (ALS). Muscle Nerve. 2020;41:107-9.

**Symptomatology and Clinical Reasoning**

An 82-year-old woman is evaluated in the office for constipation. For the past four months, she has had only one bowel movement weekly despite using an over-the-counter laxative. She sought medical care today after her constipation worsened two weeks ago and failed to respond to a laxative prescribed by her primary care physician. She is receiving outpatient care at another medical institution for hypertension, dyslipidemia, chronic kidney disease, osteoporosis, and osteoarthritis of the knees. She does not know which medications she takes and forgot to bring her medication booklet. No abnormalities were found on a colonoscopy performed two months earlier by her primary healthcare provider. She is 157 cm tall and weighs 48 kg. Her blood pressure is 124/72 mmHg, pulse is 85 beats per minute and regular, respiratory rate is 18 breaths per minute, and temperature is 36.5℃. Her abdomen is slightly distended but soft without tenderness or masses. Her liver and spleen are not palpable.

Which blood test is likely to be elevated in this patient?

(1)　CRP

(2)　FT_4_

(3)　White blood cell count

(4)　Sodium

(5)　Calcium

**Answer**

(5)　Calcium

**Commentary**

This elderly patient suffers from constipation. Her symptoms, history of chronic renal failure, and the fact that she has had outpatient care for osteoporosis, suggest she could have hypercalcemia from an activated vitamin D product associated with poor renal function. The symptoms of hypercalcemia are broad, ranging from general symptoms such as malaise and gastrointestinal symptoms, including loss of appetite, nausea, and constipation, to neurological symptoms such as disturbance of consciousness and coma. [1] Hypercalcemia lacks hallmark symptoms and tends to be overlooked but must be included in the differential diagnosis of malaise. Serum calcium levels should be measured. Common causes of hypercalcemia are cancer, primary hyperparathyroidism, and drugs (e.g., vitamin D, thiazide, theophylline, lithium). Other causative conditions include hyperthyroidism, secondary hyperparathyroidism, milk-alkali syndrome, chronic granulomatous diseases (sarcoidosis and tuberculosis), and familial hypocalciuric hypercalcemia. [2] Considering they promote intestinal calcium absorption, eldecalcitol and other vitamin D agents are often responsible for drug-related hypercalcemia in the elderly and those with poor renal function.

(1) The disease course and findings do not indicate elevated inflammatory response.

(2) Hypothyroidism is included in the differential diagnosis of chronic constipation.

(3) The patient has no symptoms suggestive of an abnormal white blood cell count.

(4) Hypernatremia and hyponatremia can cause disturbance of consciousness but do not typically cause constipation and other gastrointestinal symptoms.

(5) This is the correct answer.

**References**

1. Minisola S, Pepe J, Piemonte S, Cipriani C. The diagnosis and management of hypercalcaemia. BMJ*.* 2015;350:h2723.

2. Lafferty FW. Differential diagnosis of hypercalcemia. J Bone Miner Res. 1991;6;Suppl 2:S51-9; discussion S61. Suppl 2:S51-9.

**Disease Knowledge**

A 78-year-old man with a history of diabetes mellitus and hypertension is evaluated. He began having mild epigastric pain yesterday and was brought in by emergency medical services after experiencing a 39.5ºC fever, chills, and disturbance of consciousness. On admission, his height was 165 cm, weight was 71 kg, and his Glasgow Coma Scale was E4V4M6. The temperature was 39.1℃, pulse rate was 120/minute with a regular rhythm, blood pressure was 76/54 mmHg, and SpO_2_ was 96% on room air. The palpebral conjunctiva showed no signs of anemia, and the bulbar conjunctiva was jaundiced. The thorax was clear to auscultation, and the abdomen was tender to palpation from the epigastric area to the right upper quadrant. As fluid replacement failed to increase blood pressure, treatment with noradrenaline was started. A blood sample was sent for culture, and treatment with wide-spectrum antibiotics was initiated.

Axial and coronal sections from unenhanced abdominal computed tomography are shown here.


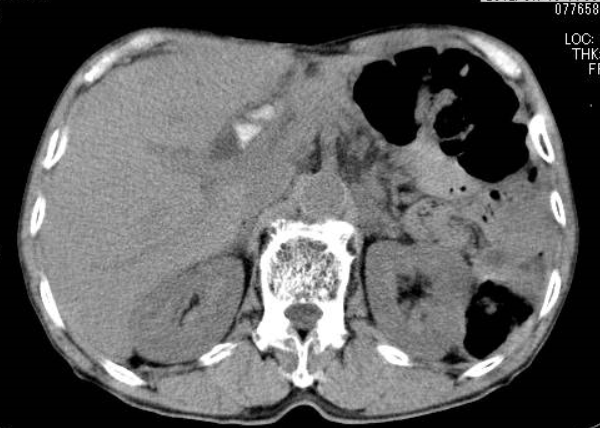


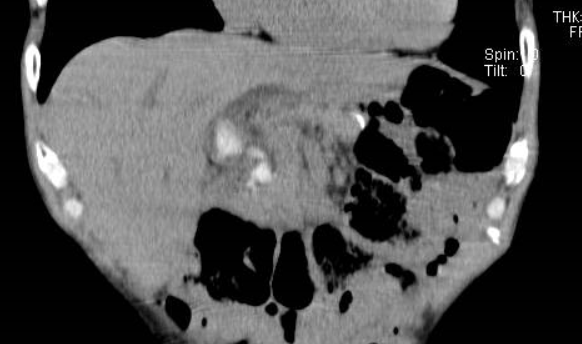


What should be done first for the management of this disease?

(1)　Endoscopic papillary balloon dilation (EPBD)

(2)　Laparoscopic choledocholithotomy

(3)　Upper gastrointestinal endoscopy

(4)　Laparoscopic cholecystectomy

(5)　Biliary drainage

**Answer**

(5)　Biliary drainage

**Commentary**

This patient has acute common bile duct stones and acute cholangitis. The CT images show biliary dilatation and several mineralized lesions in the common bile duct. The clinical finding of disturbance of consciousness and the need for vasopressors warrant a diagnosis of severe (grade III) acute cholangitis. Initial treatment for acute cholangitis is selected according to the severity assessment. Severe acute cholangitis, which can lead to circulatory failure and organ damage, requires fluid therapy, antibiotic treatment, and circulatory management but also urgent biliary drainage. The correct answer is, therefore, (5).

**Medical interview and professionalism**

You are a 2nd-year resident and saw a 58-year-old man with the chief complaint of swelling in his left calf. The physical findings were suggestive of cellulitis. You decided to give him an antibiotic. The patient mentioned that he is allergic to certain antibiotics, the names of which you did not know. Your senior resident, who was taking care of other patients, was not sure which antibiotic you would give but thought it would be fine. The pharmacist noticed that the name of the antibiotic was the generic name of an antibiotic to which the patient reported being allergic. He did not warn you since he thought you knew and still wanted to use this antibiotic. A few minutes later, the patient developed a generalized rash, respiratory distress, and hypotension consistent with anaphylactic shock.

What led to this case of medical malpractice?

(1)　You should have been more careful checking the name of the antibiotic

(2)　The senior resident should have observed your practice more carefully

(3)　The pharmacist should have questioned your decision

(4)　All of the above

(5)　This situation could not have been prevented since allergic reactions can happen, and the patient told you only generic names

**Answer**

(4)　All of the above

**Commentary**

This question involves medical malpractice, which is always a possibility lurking in routine care. Since one wrong therapeutic choice can endanger the patient's life, care must not be left to individuals but instead provided under a full system. This patient, known to be allergic to certain antibiotics, told you the wrong brand names, leading you to mistakenly believe that the antibiotic you were giving him was not one to which he was allergic. The senior resident and pharmacist failed to notice or correct this mistake, and the patient suffered anaphylactic shock as a result. This situation could have been avoided if you had more carefully determined which antibiotics the patient was allergic to, the senior resident would have involved himself in choosing the antibiotic, and the pharmacist would have questioned your decision rather than assuming everything would be all right since you are a physician. Choices 1, 2, and 3 are all correct, and it must be appreciated that multiple factors contribute to malpractice. Choice 5 is completely wrong. Most medical accidents are preventable.

**References**

1. Reason J. Human error: models and management. BMJ*.* 2000;320:768-70.
